# Supplementary material for: Epidemiology of Nocardia Species at a Tertiary Hospital in Southern Taiwan, 2012 to 2020: MLSA Phylogeny and Antimicrobial Susceptibility
Source: Antibiotics (Basel). 2022 Oct 19;11(10):1438. doi: 10.3390/antibiotics11101438 (PMC9598236; doi:10.3390/antibiotics11101438)
Supplement: Supplementary file 1 [file antibiotics-11-01438-s001.zip › antibiotics-1952660-supplementary.pdf]

Supplementary File

Table S1. Accession number for four genes of these 12 type strains of *Nocardia* species as an indicator from GenBank.

No.	Species	Strains	16S rRNA	*gryB*	*hsp65*	*secA1*
1	*Nocardia brasiliensis*	ATCC 700358^T	AY245543	GQ496125	CP003876	AY781799
2	*Nocardia cyriacigeorgica*	DSM 43005^T	AF430020	GQ496121	EF127506	GU179101
3	*Nocardia farcinica*	DSM 43665^T	OK103762	GQ496115	KF432742	GU179106
4	*Nocardia niigatensis*	DSM 44670^T	AY903615	GQ496135	AY903629	DQ360278
5	*Nocardia asteroides*	ATCC 19247^T	AY756541	GQ496120	AY756513	CP089227
6	*Nocardia beijingensis*	JCM 10666^T	NR_115827	GQ496127	KF420485	DQ360268
7	*Nocardia otitidiscaviarum*	DSM 43242^T	NR_041874	GQ496100	AY756528	CP041695
8	*Nocardia crassostreae*	ATCC 700418^T	AY756548	AB450779	AY756520	DQ360280
9	*Nocardia asiatica*	DSM 44668^T	AY903617	AB450770	AY903631	JQ773453
10	*Nocardia amikacinitoler-ans*	AEH_16681^T	MK696294	HM444068	MK886466	MK876008
11	*Nocardia concava*	DSM 44804^T	NR_115958	GQ496122	AB427103	EU178746
12	*Nocardia cerradoensis*	DSM 44546^T	AY756547	GQ496123	AY756519	KT749662

Table S2. Housekeeping genes primer.

Primer name	Sequence	Target gene	Amplicon size (bp)	PCR cycling conditions
Nocardia-E8F	AGAGTTT-GATCCTGGCTCAG	16S rRNA	499	98°C 30s, 35 cycle, 98°C 5s, 56°C 5s, 72°C 20s, 1 cycle 72°C 1min
Nocardia-534r	ATTACCGCGGCTGCTGG			
secA1-F47	GCGACGCCGAGTGGATGG	*secA1*	521	98°C 30s, 35 cycle, 98°C 5s, 63°C 5s, 72°C 20s, 1 cycle ,72°C 1min
secA1-ConR	GCGGACGATGTAGTCCTT-GTC			
gyrB-F	CTTCGCCAACAC-CATCAACAC	*gyrB*	611	98°C 30s, 35 cycle, 98°C 5s, 60°C 5s, 72°C 20s, 1 cycle, 72°C 1min
gyrB-R	TGATGATCGACTGGAC-CTCG			
hsp65-F	ACCAAC-GATGGTGTGTCCAT	*hsp65*	441	98°C 30s, 35 cycle, 98°C 5s, 54°C 5s, 72°C 20s, 1 cycle, 72°C 1min
hsp65-R	CTTGTCGAACCGCATACCCT			

[32]
